
JOURNAL INFORMATION
==============================
Journal ID: Wirtschaftsdienst

Wirtschaftsdienst

EISSN: 1613-978X

ARTICLE INFORMATION
==============================
PMCID: PMC8933190
DOI: 10.1007/s10273-022-3130-7
Article ID: 3130
Article version: 1
Subjects: Zeitgespräch

Armut in Deutschland — Einfluss der Coronapandemie noch nicht ablesbar

Poverty in Germany — Impact of Corona Pandemic Not Yet Assessable

Niehues Judith niehues@iwkoeln.de

grid.473597.b 0000 0000 9854 4639 Methodenentwicklung, Institut der deutschen Wirtschaft Köln e.V., Konrad-Adenauer-Ufer 21, 50668 Köln, Deutschland
Electronic publication date: 2022 Mar 19
Print publication date: 2022
Volume: 102
Issue: 3
First page: 181
Last page: 184
Copyright: © Der/die Autor:in 2022
License: Open Access: Dieser Artikel wird unter der Creative Commons Namensnennung 4.0 International Lizenz veröffentlicht (https://creativecommons.org/licenses/by/4.0/deed.de).
Open Access wird durch die ZBW — Leibniz-Informationszentrum Wirtschaft gefördert.
License URL: https://creativecommons.org/licenses/by/4.0/

Keywords: I32, D31
issue-copyright-statement: © The Author(s) 2022

==============================

Bereits im Frühsommer 2020 wurden mögliche Auswirkungen der Coronapandemie auf Armut und Ungleichheit diskutiert. Um erste Abschätzungen treffen zu können, wurden früh bestehende Befragungen um coronabezogene Befragungselemente ergänzt oder spezielle Ad-hoc-Befragungen durchgeführt, um beispielsweise zu beleuchten, welche Auswirkungen die Coronapandemie auf unterschiedliche Einkommens- und Erwerbsgruppen hat. Da sich die Befragungen zumeist auf bestimmte Zeitpunkte und Gruppen beziehen und auch vorliegende Simulationsanalysen mit Unsicherheiten behaftet sind, können die Ergebnisse jedoch noch kein vollständiges Bild darüber liefern, wie sich die Armutssituation beispielsweise in der Gesamtschau des ersten Pandemiejahres verändert hat. Von großem Interesse sind daher die Ergebnisse der Verteilungsindikatoren, die typischerweise – beispielsweise im Rahmen der kontinuierlichen Armuts- und Reichtumsberichterstattung – zur Bestimmung von Armut und Ungleichheit in Deutschland herangezogen werden (BMAS, 2021).

Der vorliegende Beitrag beschreibt zunächst, wie sich die Indikatoren, die konventionell zur Armutsmessung herangezogen werden, in den vergangenen Jahren in Deutschland entwickelt haben. Darauf aufbauend soll insbesondere darauf eingegangen werden, warum die Datenlage auch zum jetzigen Zeitpunkt noch keine abschließenden Folgerungen darüber erlaubt, wie sich Corona auf die Armutssituation in Deutschland ausgewirkt hat. Anhand einer Ergänzung der Betrachtung um Teilhabe- und Verschuldungsindikatoren wird zudem in aller Kürze auf die Diskussion eingegangen, warum eine isolierte Betrachtung der (relativen) Einkommenssituation nicht ausreicht, um Armutslagen umfassend bewerten und identifizieren zu können.

## Entwicklung des relativen Einkommensarmutsrisikos

Armut wird in Deutschland zumeist relativ gemessen: Gemäß amtlicher Statistik gilt eine Person als armutsgefährdet oder von Armut bedroht, wenn sie über weniger als 60 % des Medians der bedarfsgewichteten Nettoeinkommen der Gesamtbevölkerung verfügt. Trotz der häufigen Verwendung des Maßes handelt es sich bei der Armutsgefährdungsquote keineswegs um ein unumstrittenes Armutsmaß. Ein häufiger Kritikpunkt ist, dass positive Wohlstandsentwicklungen in der Quote unberücksichtigt bleiben. Dies gilt beispielsweise, wenn alle Einkommen um den gleichen prozentualen Wert steigen. Weiterhin bleibt bei der reinen Einkommensbetrachtung unberücksichtigt, ob eine Person mit einem Einkommen unterhalb der Armutsgefährdungsschwelle möglicherweise auf ein nennenswertes Vermögen zurückgreifen kann – oder umgekehrt, Personen mit Einkommen oberhalb des Schwellenwerts durch Rückzahlungsverpflichtungen belastet sind. Da die Armutsgefährdungsquote somit vorrangig ein Indikator zur Messung der Einkommensverteilung im unteren Einkommensbereich ist, sind Forschende des DIW dazu übergangen, von einer Niedrigeinkommensquote zu sprechen (Grabka et al., 2019; für eine kritische Auseinandersetzung mit dem Armutsbegriff: Brenke, 2018).

Abbildung 1 stellt die Entwicklung der Armutsgefährdungsquote auf Basis der drei Datensätze dar, die typischerweise für Armutsstatistiken in Deutschland herangezogen werden. Einzig das Sozio-oekonomische Panel (SOEP) erlaubt eine Betrachtung über einen längeren Zeitraum und verdeutlicht, dass das Armutsrisiko insbesondere zwischen 1997 und 2004 von rund 10 % auf 14 % erkennbar angestiegen ist. Auch im weiteren Verlauf zeigt sich auf Basis des SOEP ein Anstieg, der jedoch schwächer ausfällt. Dass sich die Verteilungsindikatoren im Zuge der guten Beschäftigungs- und Wirtschaftsentwicklung nicht ebenfalls positiver entwickelt haben, hängt auch mit strukturellen Veränderungen der Gesellschaft zusammen, beispielsweise mit der gestiegenen Zuwanderung und veränderten Haushaltseigenschaften, wie Auswertungen zu den Erklärungsfaktoren der Einkommensentwicklung zwischen 2005 und 2015 im Sechsten Armuts- und Reichtumsbericht der Bundesregierung gezeigt haben (Kleimann et al., 2020).

Abbildung 1

**Entwicklung der Armutsgefährdungs-/Niedrigeinkommensquote**

Anteil der Personen, die über weniger als 60 % des Medians der bedarfsgewichteten Nettoeinkommen verfügen (in realen Preisen), in %

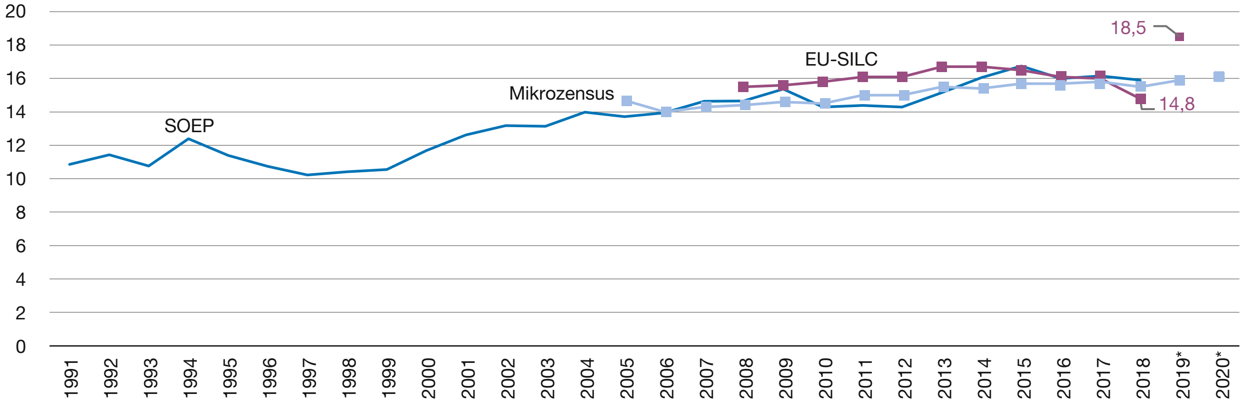

* Aufgrund einer Stichprobenumstellung ist der EU-SILC-Wert für 2019 nicht mit den Vorjahren vergleichbar, Gleiches gilt im Mikrozensus für die Ergebnisse des Jahres 2020.

Die Jahreszahlen beziehen sich jeweils auf die Einkommensinformationen der Erhebungen.

Quellen: Indikatoren des Armuts- und Reichtumsbericht, https://www.armuts-und-reichtumsbericht.de/DE/Indikatoren/Armut/Armutsrisikoquote/armutsrisikoquote.html (8. Dezember 2021); SOEP v36, eigene Berechnungen; Eurostat (EU-SILC, 2019); Amtliche Sozialberichterstattung (Mikrozensus, 2020).

Im aktuell verfügbaren Einkommensjahr 2018 liegt die Armutsgefährdungsquote auf Basis des SOEP bei 15,9 %. Die Daten gehen auf die SOEP-Erhebung des Jahres 2019 zurück, die detailliert Einkommenskomponenten des Vorjahres erfragt. Neben unregelmäßigen und regelmäßigen Arbeitseinkommen und Vermögenseinkommen wird auch der Nettomietvorteil aus selbstgenutztem Wohneigentum (*imputed rents*) als zusätzlicher Einkommensbestandteil berücksichtigt. Zudem verstehen sich die Einkommen abzüglich Steuern und Abgaben sowie zuzüglich staatlicher Transferzahlungen und privater Renten. Gemäß SOEP galt im Jahr 2018 eine alleinstehende Person als von Armut bedroht, wenn sie über weniger als 1.216 Euro netto im Monat verfügte.[1]

Mit einer Stichprobengröße von rund 1 % der gesamten Bevölkerung stellt der Mikrozensus die größte Haushaltsbefragung in Deutschland dar, in der das monatliche Haushaltseinkommen allerdings nur klassifiziert abgefragt wird. Neben der Stichprobengröße kennzeichnet sich der Mikrozensus dadurch, dass er zwar weniger detaillierte, aber dafür zeitnahe Informationen über die Einkommenssituation liefert. Zum aktuellen Zeitpunkt ist der Mikrozensus der einzige der drei abgebildeten Datensätze, der Einkommensinformationen für das Jahr 2020 und somit für (den Beginn) der Coronakrise enthält. Vor diesem Hintergrund ist man schnell geneigt, den Anstieg der Armutsgefährdungsquote von 15,9 % im Jahr 2019 auf 16,1 % im Jahr 2020 als Folge der Coronapandemie einzuordnen. Allerdings hatte im Jahr 2020 – unabhängig von der Coronapandemie – eine Neukonzeption des Mikrozensus stattgefunden (Hundenborn und Enderer, 2019). Ein Vergleich der Medianeinkommen deutet auf einen nominalen Anstieg der Armutsgefährdungsschwelle von 1.074 Euro im Jahr 2019 auf 1.126 Euro im Jahr 2020. Der rekordhafte nominale Einkommensanstieg lässt sich schwerlich mit der Rekord-Kurzarbeit und den Einkommenseinbußen im ersten Coronajahr in Einklang bringen – der Einfluss der Coronapandemie ist aus der Entwicklung somit nicht ablesbar.

## Europäische Armutsgefährdung

Von der Neukonzeption des Mikrozensus ist auch die europäische Erhebung über Einkommen und Lebensbedingungen (die sogenannte European Union Statistics on Income and Living Conditions; EU-SILC) betroffen, die in die offiziellen europäischen Armutsstatistiken von Eurostat einfließt. Während EU-SILC bisher als freiwillige Erhebung ausgestaltet war, ist das zusätzliche Fragenprogramm nun verpflichtender Bestandteil für eine Teilstichprobe des Mikrozensus. Ähnlich wie das SOEP bildet auch das EU-SILC die Einkommen im Kalenderjahr vor der Erhebung ab. Dieser Hinweis ist insbesondere vor dem Hintergrund eines möglichen Einflusses der Coronapandemie bedeutend, da die aktuell verfügbaren Armuts- und Verteilungsindikatoren des EU-SILC in den Datenbanken von Eurostat und des Statistischen Bundesamts zwar dem Jahr 2020 zugeordnet sind, sich

1 Ohne Berücksichtigung des Nettomietvorteils aus selbstgenutztem Wohneigentum läge die Schwelle bei 1.141 Euro.

die zugrundeliegenden Einkommen jedoch auf das Jahr 2019 beziehen.[2]

Einhergehend mit der Umstellung der Stichprobe erhöhte sich die Armutsgefährdungsquote gemäß EU-SILC von 14,8 % im Einkommensjahr 2018 auf 18,5 % im Einkommensjahr 2019. Ein Blick auf die Armutsgefährdungsquote in sozioökonomischen Teilgruppen deutet darauf hin, dass der Anstieg im Wesentlichen mit der besseren Abbildung der Bevölkerung mit Migrationserfahrungen zusammenhängt, die in den vorherigen Wellen des EU-SILC deutlich unterrepräsentiert war. So erhöhte sich die Armutsgefährdungsquote von Kindern, deren Eltern im Ausland geboren sind, von 13,4 % im Einkommensjahr 2018 auf 31,2 % im Jahr 2019, die der Erwachsenen, die im Ausland geboren sind, von 15,6 % auf 29,6 %. Demgegenüber haben sich die Armutsgefährdungsquoten von Kindern und Erwachsenen, die in Deutschland geboren sind, nur geringfügig erhöht (Anstiege um 0,8 respektive 0,5 Prozentpunkte). Während das Armutsrisiko von Kindern und Menschen mit Migrationshintergrund auf Basis des EU-SILC zuvor im Vergleich zum SOEP und Mikrozensus immer deutlich niedriger ausfiel, bewegen sich die Quoten nun auf einem eher ähnlichen Niveau. Unklar ist aktuell jedoch noch, warum sich auf Basis des neuen EU-SILC ein deutlich höheres allgemeines Armuts- und Ungleichheitsniveau gegenüber dem Mikrozensus ergibt, obwohl das EU-SILC Teilstichprobe desselbigen ist.

Auch wenn die verschiedenen Datensätze auf komplexe Unterschiede im Detail deuten, lassen sich zumindest auf Basis von SOEP und Mikrozensus relativ robust Risikogruppen identifizieren, die nicht nur häufiger, sondern auch dauerhafter von Armut betroffen sind. Hierzu zählen neben Arbeitslosen, insbesondere Alleinerziehende, Familien mit drei Kindern und mehr, Menschen mit Migrationshintergrund sowie Menschen mit niedrigen Bildungsabschlüssen. Demgegenüber offenbaren die Datensätze jedoch auch bedeutende Unterschiede. Während gemäß SOEP Menschen in Ostdeutschland durch ein erkennbar höheres Armutsrisiko gekennzeichnet sind, haben sich gemäß Mikrozensus die Armutsrisiken zwischen Ost- und Westdeutschland beinahe angenähert. Weiterhin verlaufen Niveau und Entwicklung der Altersarmutsrisiken im SOEP deutlich günstiger als im Mikrozensus.[3]

2 Beim Statistischen Bundesamt wird der Vorjahrsbezug durch eine entsprechende Fußnote schnell ersichtlich, in der Eurostat-Datenbank wird dieser Aspekt jedoch nur durch eine detaillierte Recherche der Metadaten erkennbar.

3 Da im SOEP auch ein aktuelles monatliches Haushaltsnettoeinkommen abgefragt wird (sogenannter *income screener*), lässt sich zeigen, dass die Unterschiede nicht auf die verschiedenen Einkommenskonzepte zurückgehen.

## Einbeziehung weiterer Dimensionen

Informationen zu Teilhabemöglichkeiten, Vermögen und Verbindlichkeiten im SOEP geben zudem Aufschluss darüber, dass sich die Betrachtung der Armutsrisiken nach Lebensphasen substanziell ändern kann, wenn diese Kriterien bei der Bewertung von Armutssituationen mit einbezogen werden. Bei dem Konzept der materiellen Deprivation wird beispielsweise untersucht, inwiefern Personen sich als üblich geltende Gebrauchsgüter nicht leisten können oder an Aktivitäten des alltäglichen Lebens aus finanziellen Gründen nicht teilhaben können. Ein Blick auf verschiedene Teilhabemöglichkeiten im SOEP 2019 verdeutlicht zunächst, dass im Jahr 2019 nur wenige Menschen in Deutschland aus finanziellen Gründen auf elementare Dinge wie angemessenes Heizen, dem Ersetzen abgetragener Kleidung oder auf einen Internetanschluss verzichten mussten. Demgegenüber galt für deutlich mehr Menschen, dass abgenutzte Möbel nicht durch neue ersetzt werden konnten (13 %) oder dass aus finanziellen Gründen nicht jedes Jahr mindestens eine einwöchige Urlaubsreise gemacht wurde (15 %). Gerade vor dem Hintergrund möglicher Einkommenseinbußen durch die Coronapandemie ist ebenso der Befund bedeutend, dass 18 % der Bundesbürger:innen in Haushalten lebten, die nicht auf finanzielle Rücklagen für Notfälle zurückgreifen konnten. Nicht nur aber gerade bei diesem Merkmal zeigen sich große Unterschiede zwischen verschiedenen Lebensphasen. Während knapp ein Viertel aller Kinder im Jahr 2019 in Haushalten ohne finanzielle Rücklagen lebte, traf dies in der Altersgruppe ab 65 Jahren auf rund ein Zehntel zu.

Auch wenn Haushalte über ein ähnliches Einkommen verfügen, können die finanziellen Spielräume sehr unterschiedlich ausfallen, je nachdem ob finanzielle Rücklagen vorliegen oder ob der Haushalt durch Rückzahlungsverpflichtungen bestehender Kredite belastet ist. Bei einer Differenzierung nach dem Einkommen zeigt sich, dass armutsgefährdete Haushalte im Durchschnitt etwas seltener durch Konsumentenkredite und durch etwas geringere Rückzahlungsverpflichtungen belastet sind als beispielsweise die Gruppe derjenigen mit einem Einkommen zwischen 60 % und 80 % des bedarfsgewichteten Medianeinkommens. Dies bekräftigt, dass eine alleinige Betrachtung eines temporären Einkommens häufig nicht ausreicht, um Armutslagen umfassend zu bestimmen. Wird neben dem Einkommen ebenfalls die Vermögenssituation mitberücksichtigt, verändert sich insbesondere die Armutsbetroffenheit älterer Haushalte, da diese häufiger auf Vermögen zurückgreifen können (Calderón et al. 2020). Hierin mag eine Erklärung für die Unterschiede in den finanziellen Sorgen liegen, die ebenfalls regelmäßig im SOEP abgefragt werden. Während sich im Jahr 2019 im Gesamtdurchschnitt rund 12 % der Erwachsenen große Sorgen um ihre eigene wirtschaftliche Situation machten, waren es in der Altersgruppe ab 65 Jahren mit knapp 7 % etwa halb so viele.

## Fazit und Ausblick

Während das Armutsrisiko zwischen dem Ende der 1990er Jahre und etwa 2004 recht deutlich angestiegen ist, zeigt sich in der Folge zwar ein weiterer Anstieg, der aber wesentlich flacher verlief als der Anstieg rund um die Jahrtausendwende. Insbesondere im Zuge der positiven Einkommensentwicklung in den Jahren vor der Coronapandemie ging die Entwicklung zudem mit einem stärkeren Anstieg der Armutsgefährdungsschwelle einher, die sich auch in einem Rückgang materieller Entbehrungen spiegelte. Im Jahr 2015 gaben noch beinahe ein Viertel der Haushalte an, dass sie in Notfällen nicht auf finanzielle Rücklagen zurückgreifen können, während der Anteil im Jahr 2019 auf 18 % zurückging.

Inwiefern die Coronapandemie Armut und Deprivation in Deutschland beeinflusst hat, lässt sich noch nicht bewerten, da die Ergebnisse des Mikrozensus 2020 aufgrund eines Zeitreihenbruchs keine Vergleiche mit den Vorjahren erlauben und sich die Einkommensangaben der 2020er Welle des EU-SILC auf das Kalenderjahr 2019 beziehen. Auch mit der in Kürze zu erwartenden SOEP-Welle-2020 lässt sich der Einfluss der Coronapandemie auf das Jahr 2020 noch nicht abschließend beurteilen, da sich deren detaillierte Einkommensabfragen ebenfalls auf das Jahr 2019 beziehen und gerade für die Bewertung der Einkommensveränderungen durch die Coronakrise die Berücksichtigung unregelmäßiger Einkommen bedeutend ist.

Dass sich die Armutsgefährdungsquote nach der Stichprobenumstellung im Mikrozensus im Jahr 2020 weiterhin auf einem Niveau von rund 16 % bewegt, deutet jedoch darauf hin, dass sich das relative Einkommensarmutsrisiko im Zuge der Coronapandemie möglicherweise nicht stark verändert hat. Simulationsanalysen der Einkommenseffekte der Coronakrise für das Jahr 2020 weisen in eine ähnliche Richtung und legen nahe, dass die Coronapandemie wahrscheinlich eine Erhöhung der Ungleichverteilung der Bruttoeinkommen impliziert, sich die Verteilung der verfügbaren Einkommen insgesamt aber wenig verändert (Bruckmeier et al., 2021). Die Simulationsergebnisse zeigen zudem, dass sich im Jahr 2020 sogar ein leichter Rückgang des statistischen Armutsrisikos ergeben könnte, da gemäß der simulierten Einkommensveränderungen die Medianeinkommen prozentual stärker zurückgingen als die Einkommen unterhalb der Armutsgefährdungsschwelle (Beznoska et al., 2020).

Auch wenn Simulationsergebnisse typischerweise mit Unsicherheiten behaftet sind, deutet sich hier erneut an, dass eine relative Einkommensbetrachtung nicht ausreicht, um Armutslagen umfassend zu identifizieren – denn auch wenn die Effekte auf die Einkommensverteilung noch nicht abschließend geklärt sind, werden Einbußen für diejenigen besonders schwer zu verkraften sein, die nicht auf finanzielle Rücklagen zurückgreifen können. Wie sich materielle Entbehrungen im Jahr 2020 verändert haben, darüber wird die kommende SOEP-Welle zeitnah Aufschluss geben können.

While the risk of poverty increased quite markedly around the turn of the millennium, a further but flatter increase can be subsequently observed. The development was also accompanied by a stronger increase in the at-risk-of-poverty threshold, particularly in the wake of the positive income trend in the years prior to the coronavirus pandemic. The extent to which the corona pandemic influenced poverty cannot yet be assessed, as the results published so far are not comparable with previous years due to a break in the available time series. However, first indications of simulation studies suggest that the relative risk of poverty and the distribution of disposable income may have changed only slightly.

Dr. Judith Niehues ist Leiterin der Forschungsgruppe Mikrodaten und Methodenentwicklung des Instituts der deutschen Wirtschaft.


Literatur

Brenke K Armut: vom Elend eines Begriffs Wirtschaftsdienst 2018 98 4 260 266 10.1007/s10273-018-2284-9
Beznoska, M., J. Niehues und M. Stockhausen (2020), Stabil durch die Krise? Verteilungsfolgen der Corona-Pandemie — eine Mikrosimulationsanalyse, IW-Report, (65), 29.
BMAS — Bundesministeriums für Arbeit und Soziales (2021), Armuts- und Reichtumsbericht, https://www.armuts-und-reichtumsbericht.de/DE/Indikatoren/Armut/Armutsrisikoquote/armutsrisikoquote.html (25. Februar 2022).
Bruckmeier, K., B. Fitzenberger, J. Wiemers (2021), Corona führte zwar bisher nicht zu gestiegener Einkommensungleichheit — für eine Entwarnung ist es dennoch zu früh, IAB-Forum, 7. Oktober, https://www.iab-forum.de/corona-fuehrte-zwar-bisher-nicht-zu-gestiegener-einkommensungleichheit-fuer-eine-entwarnung-ist-es-dennoch-zu-frueh/ (24. Februar 2022).
Calderón M Niehues J Stockhausen M Wie verteilt sich der Wohlstand in Deutschland? Eine kombinierte Betrachtung von Einkommen und Vermögen IW-Trends 2020 47 3 39 60
Grabka M Goebel J Liebig S Wiederanstieg der Einkommensungleichheit — aber auch deutlich steigende Realeinkommen DIW-Wochenbericht 2019 86 19 344 353
Hundenborn, J. und J. Enderer (2019), Die Neuregelung des Mikrozensus ab 2020, WISTA — Wirtschaft und Statistik, (6), 9–18.
Kleimann, R., M. Biewen, M. Sturm, A. Peichl, J. Späth, N. Laub et al. (2020), Analyse der Einkommens- und Vermögensverteilung in Deutschland. Bundesministerium für Arbeit und Soziales (Hrsg.), Forschungsbericht.
